# Genomic landscape of clinically advanced *KRAS* wild-type pancreatic ductal adenocarcinoma

**DOI:** 10.3389/fonc.2023.1169586

**Published:** 2023-06-19

**Authors:** Prashanth Ashok Kumar, Serenella Serinelli, Daniel J. Zaccarini, Richard Huang, Natalie Danziger, Tyler Janovitz, Alina Basnet, Abirami Sivapiragasam, Stephen Graziano, Jeffrey S. Ross

**Affiliations:** ^1^Upstate Cancer Center, Upstate Medical University, Syracuse, NY, United States; ^2^Department of Pathology, Upstate Medical University, Syracuse, NY, United States; ^3^Foundation Medicine, Cambridge, MA, United States

**Keywords:** pancreatic ductal adenocarcinoma, *KRAS* mutation, *KRAS* wild-type pancreatic cancer, genomic alterations, targeted therapy

## Abstract

**Introduction:**

KRAS mutation is a common occurrence in Pancreatic Ductal Adenocarcinoma (PDA) and is a driver mutation for disease development and progression. KRAS wild-type PDA may constitute a distinct molecular and clinical subtype. We used the Foundation one data to analyze the difference in Genomic Alterations (GAs) that occur in KRAS mutated and wild-type PDA.

**Methods:**

Comprehensive genomic profiling (CGP) data, tumor mutational burden (TMB), microsatellite instability (MSI) and PD-L1 by Immunohistochemistry (IHC) were analyzed.

**Results and discussion:**

Our cohort had 9444 cases of advanced PDA. 8723 (92.37%) patients had KRAS mutation. 721 (7.63%) patients were KRAS wild-type. Among potentially targetable mutations, GAs more common in KRAS wild-type included ERBB2 (mutated vs wild-type: 1.7% vs 6.8%, p <0.0001), BRAF (mutated vs wild-type: 0.5% vs 17.9%, p <0.0001), PIK3CA (mutated vs wild-type: 2.3% vs 6.5%, p <0.001), FGFR2 (mutated vs wild-type: 0.1% vs 4.4%, p <0.0001), ATM (mutated vs wild-type: 3.6% vs 6.8%, p <0.0001). On analyzing untargetable GAs, the KRAS mutated group had a significantly higher percentage of TP53 (mutated vs wild-type: 80.2% vs 47.6%, p <0.0001), CDKN2A (mutated vs wild-type: 56.2% vs 34.4%, p <0.0001), CDKN2B (mutated vs wild-type: 28.9% vs 23%, p =0.007), SMAD4 (mutated vs wild-type: 26.8% vs 15.7%, p <0.0001) and MTAP (mutated vs wild-type: 21.7% vs 18%, p =0.02). ARID1A (mutated vs wild-type: 7.7% vs 13.6%, p <0.0001 and RB1(mutated vs wild-type: 2% vs 4%, p =0.01) were more prevalent in the wild-type subgroup. Mean TMB was higher in the KRAS wild-type subgroup (mutated vs wild-type: 2.3 vs 3.6, p <0.0001). High TMB, defined as TMB > 10 mut/mB (mutated vs wild-type: 1% vs 6.3%, p <0.0001) and very-high TMB, defined as TMB >20 mut/mB (mutated vs wild-type: 0.5% vs 2.4%, p <0.0001) favored the wild-type. PD-L1 high expression was similar between the 2 groups (mutated vs wild-type: 5.7% vs 6%,). GA associated with immune checkpoint inhibitors (ICPIs) response including PBRM1 (mutated vs wild-type: 0.7% vs 3.2%, p <0.0001) and MDM2 (mutated vs wild-type: 1.3% vs 4.4%, p <0.0001) were more likely to be seen in KRAS wild-type PDA.

## Introduction

Pancreatic Ductal Adenocarcinoma (PDA) is the most common pancreatic neoplasm comprising 90% of the malignancies arising from the organ (1). The incidence of PDA has been on the rise over the past decade and is projected to be the second leading cause of cancer related mortality in the United States by 2030. Despite the improvement in 5-year survival from 5.26% in 2000 to nearly 10% in 2020, the overall prognosis remains grim (2). The median survival of patients with PDA is only 3-5 months despite all the advances in therapy, necessitating the need for further research (3).

Somatic mutation testing for alterations like *BRCA, KRAS, HER2, PALB2* and mismatch repair proteins, for PDA patients is recommended in the NCCN guidelines (4). Around 10% of PDA patients express alterations that are potentially targetable (5). PDA can be highly heterogenous in both biology and clinical behavior (6). *KRAS* mutation is a common occurrence in PDA and is identified in more than 90% of the sequenced cases. *KRAS* mutation occurs early in the development of PDA and is a key oncologic driver in this disease (7). *KRAS* wild-type PDA, although relatively less frequent, appears to have distinctive characteristics, both genetically and clinically. Treatment resistant PDA may feature selection of *KRAS* wild-type cancer cells that confer a survival advantage, making them more aggressive and resistant to targeted therapy (8). Thus, *KRAS* mutated and wild-type PDA may represent contrasting molecular subtypes and this distinction has important implications in assessing patients’ clinical course (7, 9). Their response to treatments like immune checkpoint inhibitors (ICPIs) therapy may also be different, as *KRAS* mutations have shown to affect the immune-microenvironment (10). Modern approaches for the treatment of PDA have moved towards personalized care and identifying therapeutic molecular targets that have the potential to impact the selection of treatment lines (11). This is evident from the results of the recently published results of CodeBreaK 100, that solidified the anti-cancer activity of sotorasib in this disease (12). In this study, we utilized a large database (13), to provide a descriptive analysis of the differences in genomic alterations (GAs) between *KRAS* mutated and wild-type PDAs (14) and characterize the GAs that occur in the less common *KRAS* wild-type PDAs.

## Methodology

### General

Approval for this study, including a waiver of informed consent, was obtained from the Western Institutional Review Board (Protocol No. 20152817). A retrospective database search of a CLIA-certified and CAP-accredited reference molecular laboratory was performed for 9,444 PDA tissue samples. All PDA cases were clinically advanced, either inoperable or metastatic. The patient age and gender, routine histology and immunohistochemical staining results and confirmation of the diagnosis, were extracted from medical records and pathology reports. All PDA cases submitted to Foundation Medicine and sequenced using the FoundationOne CDx assay from January 1, 2018, to December 31, 2020, were eligible for inclusion in this study. Only cases with adequate tissue sample size, DNA extraction amounts of 50 ng or greater, tissues with a minimum of 20% tumor nuclear area versus benign nuclear area either before or after pathologist-guided macro-enrichment were included. In addition, cases with low tumor purity on sequencing or inadequate sequencing coverage depth as described in the FoundationOne CDx US FDA approval were excluded from the study (14).

### Sample sequencing

Comprehensive genomic profiling (CGP) of all 9,444 PDA FFPE tissue samples was performed on extracted DNA using hybridization-capture- adaptor ligation–based libraries (FoundationOneCDx, Foundation Medicine, Inc.). All samples forwarded for DNA extraction contained a minimum of 20% tumor nuclei. The samples were assayed using all coding exons from 324 cancer related genes, plus select introns from at least 31 genes frequently rearranged in cancer. The PDA specimens were evaluated for all classes of GAs including base substitutions, insertions, deletions, copy number alterations (amplifications and homozygous deletions), and for select gene fusions/rearrangements, as previously described (14–16). The bioinformatics processes used in this study included Bayesian algorithms to detect base substitutions, local assembly algorithms to detect short insertions and deletions, a comparison with process-matched normal control samples to detect gene copy number alterations and an analysis of chimeric read pairs to identify gene fusions as previously described. To help visualize the sequencing data results, an oncoprint plot was generated with the online tools of the cbio portal as described by Gao et al (17) and Cerami et al (18). Tumor mutational burden was determined on 0.83–1.14 Mb of sequenced, as previously described. In this study, mutational burden scores were defined by mutation/Mb. Assessment of microsatellite instability was performed from DNA sequencing at least 95 loci, as previously described. Each microsatellite locus had repeat length of 7–39 bp. The next-generation sequencing based “microsatellite instability score” was translated into categorical “microsatellite instability high”, “microsatellite instability intermediate”, or “microsatellite stable” by unsupervised clustering of specimens for which microsatellite instability status was previously assessed *via* gold standard methods (19, 20).

### PD-L1 immunohistochemistry

PD-L1 expression was determined on subsets of the tumors using the DAKO 22C3 assay with low-positive tumor cell scoring defined as 1%-49% staining and high-positive tumor cell scoring defined as 50% staining (21). Anti-PD-L1 staining was done using the Dako 22C3 IHC kit, following the instructions provided in the kit protocol. Results were scored using the widely used tumor proportional score system (TPS) (22).

### Statistical analysis

Chi-square test and Mann Whitney U test were used in the statistical comparisons of the 2 groups. Statistical significance was defined as p < 0.05.

## Results

8723/9444 (92.37%) of the PDA featured *KRAS* mutation. 721 (7.63%) patients were *KRAS* wild-type. Males were more prevalent in both groups [*KRAS* mutated: 52/48% (M/F), *KRAS* wild-type 61/39% (M/F)]. The median age was similar in both groups [*KRAS* mutated: 67 years, KRAS wild-type: 65 years]. The average GA/tumor was 4.88 for *KRAS* mutated and 4.47 for *KRAS* wild type (**Table 1**).

**Table 1:** Descriptive analysis of the patient characteristics and various GAs.

	KRAS Wild-type	KRAS Mutated	P Value
**Cases**	721	8,723	
**Males/Females**	61%/39%	52%/48%	<.0001
**Median age**	65	67	NS
**Mean age**	63.7	65.9	<.0001
**GA/tumor**	4.47	4.88	NS
Top Untargetable GA			
***TP53***	47.6%	80.2%	<.0001
***CDKN2A***	34.4%	56.2%	<.0001
***CDKN2B***	23.0%	28.9%	=.0007
***SMAD4***	15.7%	26.8%	<.0001
***MTAP***	18.0%	21.7%	=.02
***CDK6***	1.9%	2.7%	NS
***ARID1A***	13.6%	7.7%	<.0001
***RB1***	4.0%	2.0%	=.001
Top Potentially Targetable GA			
***EGFR SV***	<1%	<1%	NS
***ERBB2***	6.8%	1.7%	<.0001
***ALK Fusion***	1.0%	0%	NS
***BRAF***	17.9%	0.5%	<.0001
***PIK3CA***	6.5%	2.3%	<.0001
***FGFR1***	1.2%	1.6%	NS
***FGFR2***	4.4%	0.1%	<.0001
***PTEN***	7.2%	1.2%	<.0001
***KRAS G12C***	0%	1.6%	<.0001
***BRCA1***	1.8%	1.2%	NS
***ATM***	6.8%	3.6%	<0001
***BRCA2***	2.5%	2.9%	NS
ICPI Predictive GA			
***PBRM1***	3.2%	0.7%	<.0001
***STK11***	3.3%	2.5%	NS
***MDM2***	4.4%	1.3%	<.0001
***CD274 amp***	<1%	<1%	NS
ICPI Predictive Biomarkers			
**MSI-High**	1.7%	<0.9%	NS
**Mean TMB**	3.6	2.3	<.0001
**Median TMB**	2.5	1.3	
**TMB>10mut/mB**	6.3%	1%	<.0001
**TMB>20mut/Mb**	2.4%	<0.5%	<.0001
**PD-L1 IHC Low+**	21.8% (257)	31.8% (3069)	=.0007
**PD-L1 IHC High+**	6.0%	5.7%	NS

The descriptive analysis of the various GAs in our cohort, divided into targetable and untargetable GAs is shown in **Table 1**. Among potentially targetable mutations, GAs more common in *KRAS* wild-type included *ERBB2* (mutated vs wild-type: 1.7% vs 6.8%, p <0.0001), *BRAF* (mutated vs wild-type: 0.5% vs 17.9%, p <0.0001), *PIK3CA* (mutated vs wild-type: 2.3% vs 6.5%, p <0.001), *FGFR2* (mutated vs wild-type: 0.1% vs 4.4%, p <0.0001), *ATM* (mutated vs wild-type: 3.6% vs 6.8%, p <0.0001). *KRAS G12C* comprised 1.6% of the mutated group. On analyzing untargetable GAs, the *KRAS* mutated group had a significantly higher percentage of *TP53* (mutated vs wild-type: 80.2% vs 47.6%, p <0.0001), *CDKN2A* (mutated vs wild-type: 56.2% vs 34.4%, p <0.0001), *CDKN2B* (mutated vs wild-type: 28.9% vs 23%, p =0.007), *SMAD4* (mutated vs wild-type: 26.8% vs 15.7%, p <0.0001) and *MTAP* (mutated vs wild-type: 21.7% vs 18%, p =0.02). *ARID1A* (mutated vs wild-type: 7.7% vs 13.6%, p <0.0001) and *RB1 (*mutated vs wild-type: 2% vs 4%, p =0.01) were more prevalent in the wild-type subgroup.

Markers predictive of ICPIs therapy response were studied and favored the wild-type subgroup. Mean tumor mutational burden *(*TMB*)* was higher in the wild-type subgroup (mutated vs wild-type: 2.3 vs 3.6, p <0.0001). High TMB, defined as TMB > 10 mut/mB (mutated vs wild-type 1% vs 6.3%, p <0.0001) and ultra-high TMB, defined as TMB >20 mut/mB (mutated vs wild-type: 0.5% vs 2.4%, p <0.0001) favored the wild-type. *PD-L1* high was similar between the 2 groups (mutated vs wild-type: 5.7% vs 6%). GAs predictive of immune checkpoint therapy (IO) response like *PBRM1* (mutated vs wild-type: 0.7% vs 3.2%, p <0.0001) and *MDM2* (mutated vs wild-type: 1.3% vs 4.4%, p <0.0001) were more likely to be seen in wild-type subtype.

**Figure 1:**
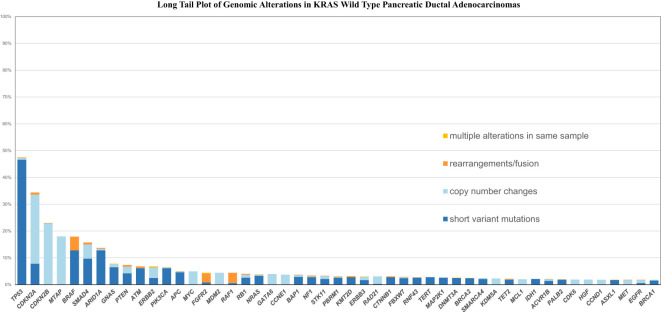
Long tail plot of GA in *KRAS* Wild-Type *PDA*.

**Figure 2:**
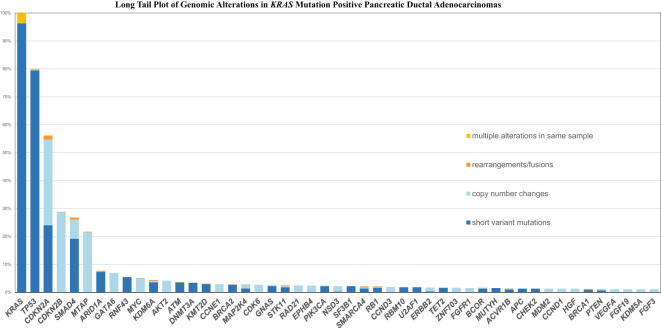
Long Tail Plot of GA in *KRAS* mutated PDA.

**Figures 1**, **2** shows the long tail plots of the various GA in *KRAS* wild-type and *KRAS* mutated PDA respectively.

**Figure 3:**
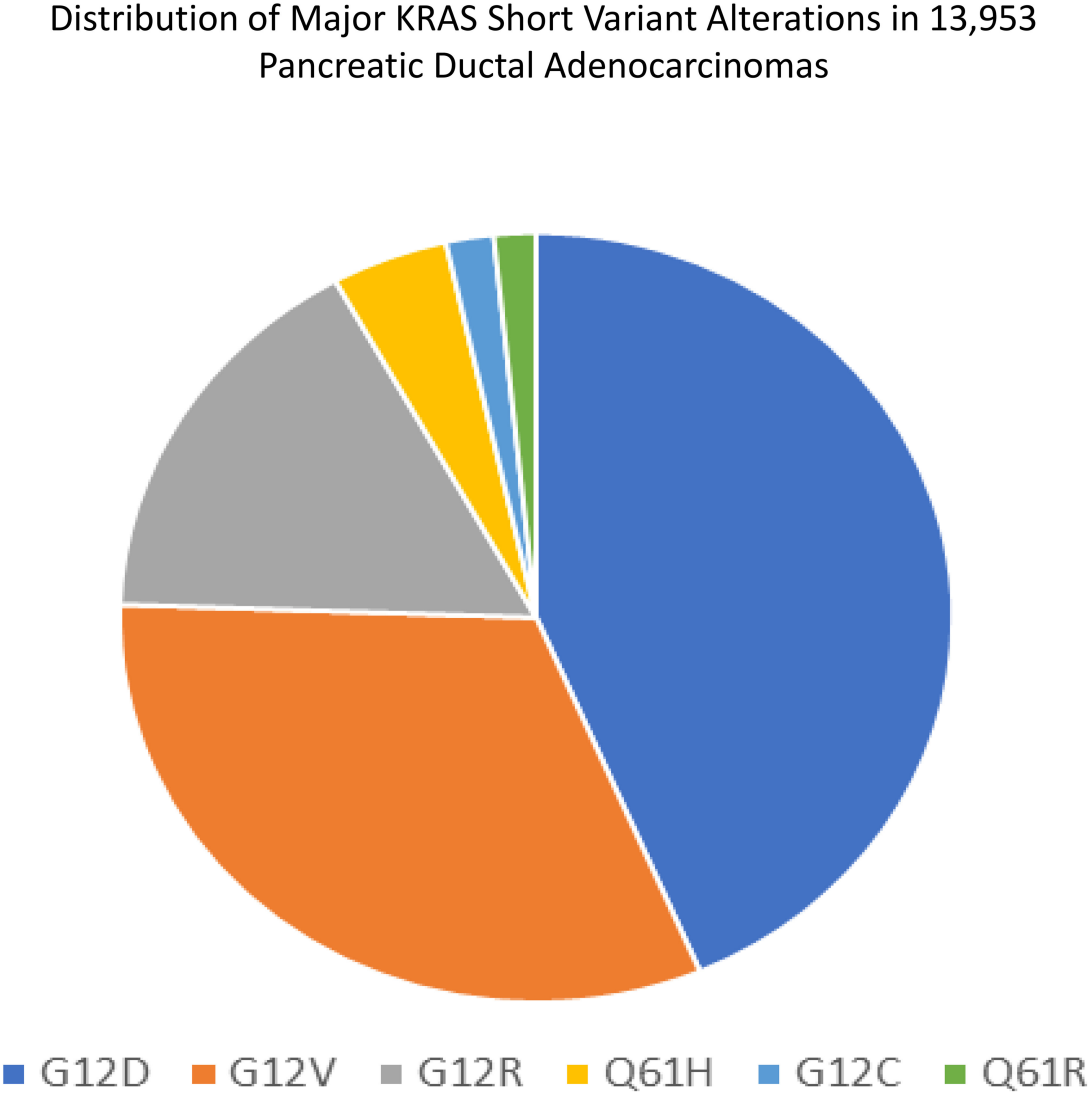
Distribution of major *KRAS* short variant alterations in 13,953 PDAs. Sample size is different from the cohort used in our study and includes earlier versions of the Foundation One CDx test with different/smaller bait sets.

**Figure 3** demonstrates the distribution of the various *KRAS* short variant alterations. Sample size is different from the cohort used in our study and includes earlier versions of the Foundation One CDx test with different/smaller bait sets.

**Figure 4:**
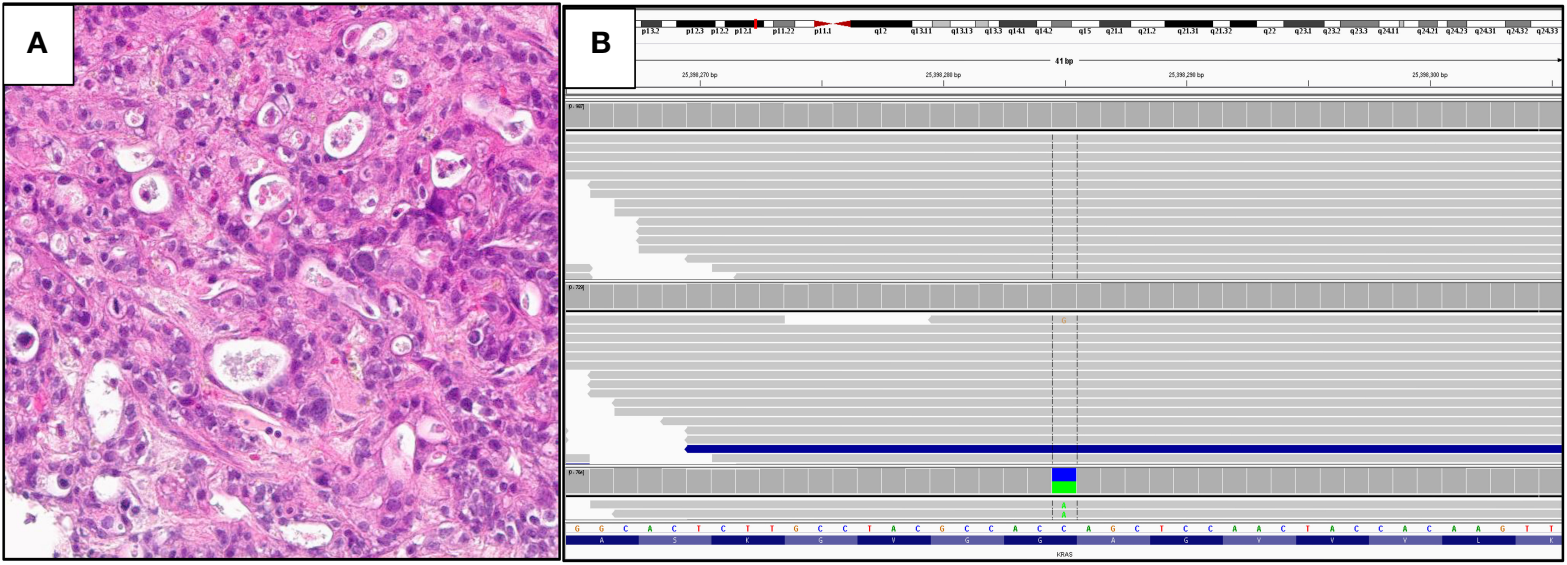
**(A)** Hematoxylin and eosin: histology of the tumor. **(B)** IGV View of KRAS G12C mutation.

**Figure 4** shows a case of metastatic PDA to the liver in a 51-year-old man. The tumor was positive for CK7 and CA19-9 and negative for CK20, HAS and TTF1. On comprehensive genomic profiling this tumor was microsatellite (MS) stable and featured a TMB of 8 mutations/MB. It had a *KRAS G12C* mutation along other short variant mutations in *CDKN2A/B* and *TP53* along with *MDM2* amplification.

**Figure 5:**
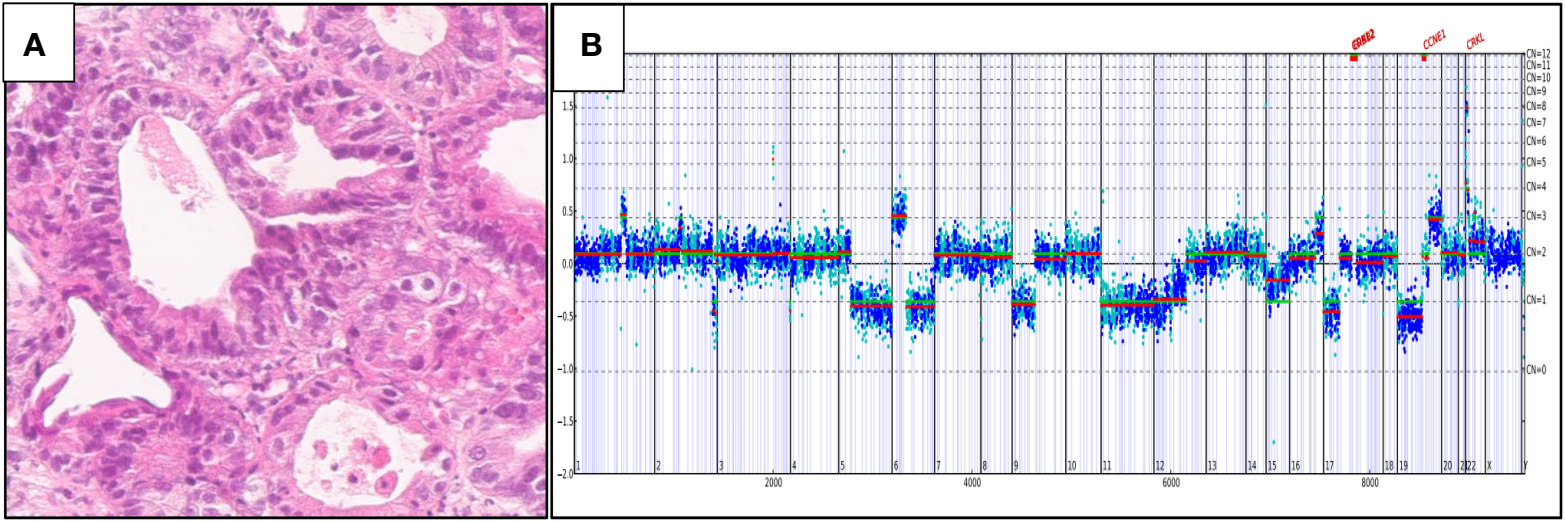
**(A)** Hematoxylin and eosin: histology of the tumor. **(B)** ERBB2 amplification at 148 copies.

**Figure 5** shows a case of metastatic PDA to the liver in a 75-year-old woman. This tumor stained positively for CK7 and CA19-9 and negatively for CK20 and CDX2. On comprehensive genomic profiling, this tumor was *KRAS* wild-type, MS stable and featured a low TMB of 1 mutation/Mb. There was major *ERBB2* amplification at 148 copies along with APC E468*, MAP2K4 Q316* and *TP53 C275F* short variant mutations, CCNE*1* and *CRKL* amplification and a *CDK12* inversion exons 8-11.

**Figures 4B**, **5B** were generated by the Foundation Medicine “Curation Analysis Tool Interface” linked directly to the Integrative Genomics Viewer (IGV) developed by the Broad Institute of the Massachusetts Institute of Technology (23). All the GA and mutations seen in both the cohorts are available in the supplement (S1: *KRAS* Wild-Type, S2: *KRAS* mutated).

## Discussion

In our study of PDAs, 93.37% had *KRAS* mutation, whereas 7.63% were wild-type. This is similar to other studies in literature where *KRAS* mutation is noted in around in 85-90% of pancreatic cancers (7, 24). The slightly higher frequency of *KRAS* mutations in the study cohort may reflect that all of the patients had relapsed and refractory disease that was predominantly metastatic at the time of sequencing reflecting the adverse prognostic influence associated with *KRAS* mutation in PDA. Epidemiologic characteristics of our cohort were consistent with those established for pancreatic cancer, showing male preponderance and older age (25). *KRAS* mutated PDA has been associated with a worse overall survival and may respond poorly to gemcitabine based therapy (26). Wild-type *KRAS* is reported in 8-12% of PDAs (27, 28). The possibility that this group may have improved survival, increases the importance to identify potential targets for precision therapies that could potentially increase both the progression-free, and the overall survivals for these patients (28). Voutsadakis et al., used publicly available genomic cohorts (The Cancer Genome Atlas, the MSK-IMPACT study, the pancreatic cancer sub-cohort of a pan-cancer study from China, and the pancreatic cancer cohort from the American Association for Cancer Research project GENIE), along with cBioportal and OncoKB knowledgebase, to characterize the GAs that occur in PDA without commonly seen alterations such as KRAS, TP53, CDKN2A and SMAD4. PDA without these alterations constituted 5-10%. No major differences in GAs were noted. The relatively limited sample size in the wild-type PDA group could be an impediment to adequate analysis in such studies, including our analysis (29).

The treatment of locally advanced, unresectable, and metastatic PDA is centered around using various chemotherapeutic regimes. Olaparib and Rucaparib, which are poly (adenosine diphosphate–ribose) polymerase (*PARP*) inhibitors, are FDA approved in the 2^nd^ line setting and can also be utilized for maintenance in patients with germline *BRCA1* and *BRCA2* mutations. There was benefit in terms of progression free survival (PFS), but overall survival remained unchanged (30, 31). Approvals such as pembrolizumab for TMB>10 and Entrectinib and Larotrectinib for *NTRK* fusions can be utilized in PDA. Overall, the utility of non-chemotherapeutic systemic treatments remains limited (4). PDA is a complex disease in terms of molecular and epigenetic changes, in which *KRAS* plays a central role from origin to progression (32). Efforts at targeting *KRAS* in pancreatic cancer have been unsuccessful so far. The mitogen-activated protein kinase (*MAPK*) pathway is an important pathway interconnected with *KRAS*. Attempts to target this with *MAPK* inhibitors either with or without gemcitabine unfortunately did not reveal any survival benefit (33). Blocking MEK and PI3K/AKT using selumetinib was also not useful to improve survival (34). *KRAS* vaccines like *RAS* peptide vaccines and those targeting mutant *KRAS* were unsuccessful (28, 35). Tipifarnib, a drug that targets *RAS* dependent growth failed to show any benefit in phase 3 trials (36). Several agents have shown promising results in PDA cell lines but the results remain exploratory (28). Sotorasib is a small molecule irreversible inhibitor of *KRAS G12C*. The CodeBreaK100 showed encouraging activity of this drug in advanced malignancies with *KRAS G12C* mutation (37). *KRAS G12C* is seen in 1-2% of patients with KRAS mutated PDA. The results from our cohort were consistent with this (1.6%). The efficacy and safety of sotorosib in pretreated *KRAS G12C* mutated PDA was reported in ASCO 2022. The overall response rate was 21.1% from a cohort of 38 patients (38).

*KRAS* wild-type PDA had a higher frequency of ERBB2 genomic alterations (6.8%). Clinical studies of anti-HER2 drugs in PDA have previously focused on *ERBB2* amplification. A phase 2 study of traztuzumab, cetuximab and gemcitabine as a first line strategy in metastatic PDA showed disease stabilization in 27% of 33 patients. Cetuximab and traztuzumab combination has also been studied (39). Although the aforementioned studies were small, the durable activity of the antibody drug conjugate, Trastuzumab deruxtecan is well established. It is approved in Non-small cell lung cancer (NSCLC) (55% objective response among 91 patients) based on the DESTINY-Lung01 for patients with ERBB2 kinase domain mutations (40) and in metastatic breast cancer for patients with HER2 low expression by IHC (41). Trials are underway to study the role of Trastuzumab deruxtecan in solid tumors including PDA (42). *BRAF* mutation was significantly higher in wild-type PDA (17.9%). This can be anticipated as *BRAF* and *KRAS* are mutually exclusive in most cancers (43, 44). The FDA has approved the combination of dabrafenib and trametinib in patients with metastatic or unresectable sold tumors with *BRAF V600E* mutation. They must have progressed through prior lines of treatment and must have no other alternative options. This was based on the Phase 2 ROAR basket study and the NCI-MATCH ECOG-ACRIN Trial (EAY131) Subprotocol Z1F (45–47). *BRAF* mutation is historically found in 3% of PDA and individual reports have shown good response with *BRAF* and *MEK* inhibitor combination (48). *PIK3CA* (6.5%), *FGFR2* (4.4%) and *ATM* (6.8%) were the other targetable GAs that were significantly higher in wild-type PDA. Increased *Akt* signaling is an important effector in the *PIK3* pathway. Activating *PIK3CA* mutations in PDA have been reported in the range of 4% in the past. Several trials evaluating various agents targeting the *PIK3* pathway are currently underway, but most of them are early phase (I/II) (49). *FGFR* alterations are reported in only 4-6% of PDAs. The only approval for *FGFR* inhibitors are in metastatic urothelial carcinoma (erdafitinib) and in cholangiocarcinoma (pemigatinib) (50, 51). A recent case report showed durable response of >12 months with erdafitinib in a young male with relapsed *FGFR* rearranged PDA. Incidentally, the patient in that particular case was also *KRAS* wild-type (52). The authors mention that the pathology revealed poorly differentiated adenocarcinoma, raising the possibility of it being a cholangiocarcinoma, as FGFR GA is more common in biliary tract cancers (52, 53). *FGFR2* GAs, which are mostly fusions, are found in 10-15% of intrahepatic cholangiocarcinoma’s (53), compared to 4.4% in our *KRAS* wild-type PDA cohort. However, the pathologic diagnosis in these cases could be questioned, raising the suspicion that these could be cholangiocarcinoma. *ATM* mutations are a part of the *HRD* germline spectrum and may connote sensitivity to *PARP* inhibitors. Found in 1.7-3.3% of all PDAs, these are significantly more common in *KRAS* wild-type and the results of early phase clinical trials pertaining to this would have to be observed (54).

On analyzing the data on markers predictive of ICPIs therapy response, several factors (higher mean TMB, higher percentage of high and ultrahigh TMB) suggests that, as a group, the *KRAS* wild-type PDA may be more responsive to ICPIs therapy than the *KRAS* mutated PDA. Other predictors of ICPIs response like PBRM1 (55, 56) and MDM2 (57) were also higher, necessitating the need for further research into this aspect.

Limitations of our study include the retrospective nature and the confounding that may arise from it. The lack of clinical outcomes data for our patients is another limitation. Subgroup analysis and stratification of PDA by *KRAS* mutation status in randomized clinical trials can help overcome this. Some cases like those with FGFR2 alterations could have been intrahepatic cholangiocarcinoma and may have been improperly diagnosed as PDA.

Overall, several targetable GAs and ICPIs positive predictive markers were more frequent in *KRAS* wild-type *PDA*. This combined with the fact that *TP53* alterations were much lower in *KRAS* wild-type PDA provides sound evidence that this distinct molecular subtype of PDA has the potential to achieve a much better survival outcome. The presence of *TP53* alteration in itself is an adverse prognostic marker in any malignancy (58) and long term PDA survivors have consistently demonstrated absence of *KRAS* and *TP53* mutations (59, 60).

## Conclusion

*KRAS* wild-type PDA has a higher frequency of several targetable GAs and may thus provide more options for targeted treatments. Response to ICPIs therapy may be better, given more patients in this group had high TMB. Overall, lower frequency of *TP53* mutation suggests that physicians and academia should strive to achieve longer survivals in *KRAS* wild-type PDA patients. Lack of prospective clinical outcome data is a limitation of our study.

## Data availability statement

The original contributions presented in the study are included in the article/supplementary material. Further inquiries can be directed to the corresponding author.

## Ethics statement

The studies involving human participants were reviewed and approved by Western Institutional Review Board (Protocol No. 20152817). Written informed consent for participation was not required for this study in accordance with the national legislation and the institutional requirements.

## Author contributions

PA: Conceptualization, Formal analysis, Validation, Visualization, Roles/Writing - original draft, Writing - review and editing. SS: Conceptualization, Data curation, Formal analysis, Funding acquisition, Investigation, Methodology, Project administration, Resources, Software, Visualization, Writing - review and editing. DZ: Conceptualization, Data curation, Formal analysis, Funding acquisition, Investigation, Methodology, Project administration, Resources, Software, Visualization, Writing - review and editing. RH: Conceptualization, Data curation, Formal analysis, Funding acquisition, Investigation, Methodology, Project administration, Resources, Software, Visualization, Writing - review and editing. ND: Conceptualization, Data curation, Formal analysis, Funding acquisition, Investigation, Methodology, Project administration, Resources, Software, Visualization, Writing - review and editing. TJ: Conceptualization, Data curation, Formal analysis, Funding acquisition, Investigation, Methodology, Project administration, Resources, Software, Visualization, Writing - review and editing. AB: Formal analysis, Validation, Visualization, Writing - review and editing. AS: Formal analysis, Validation, Visualization, Writing - review and editing. SG: Formal analysis, Resources, Validation, Visualization, Writing - review and editing. All authors contributed to the article and approved the submitted version.
